# Indiana State Dental Society

**Published:** 1882-08

**Authors:** 


### EDITORIAL.

### INDIANA STATE DENTAL SOCIETY.

The annual meeting of this body was held in Indianapolis, June 27th to 30th. The meeting was largely attended, a greater number being present than ever before.

The meeting was held in the rooms of the dental college, which were commodious and pleasant.

The occasion was one of much interest, this result was attained chiefly through the effort of the Executive Committee.

Several very excellent papers were read, two of which are found in this number of the REGISTER.

The influence of the law, regulating the practice of dentistry in Indiana is good, as was shown by the report of the Board of Examiners, by which it appears that a large proportion of those who came forward for examination and a certificate to practice are rejected by the Board. This indicates faithfulness on the part of the Board, and augurs well for the profession and the public.

A resolution was presented and passed forbidding any dealer in, or manufacture of dental goods or materials, to present or speak of them before the society without explicit permission.

While under some circumstances such a resolution might not be objectionable; it was thought by many that, as it was then presented, the proceeding was unfortunate, inasmuch as any one at any time presuming to transgress could be stopped at once.

Some matters of a more serious character came before the society for definite and prompt action. Three members who had heretofore stood well in the society, had all been members for several years, and one almost, if not quite, from the organization of the society: he had a standing and a reputation in the society and in the profession of which any one might well be pround; he was honored wherever known. Against these three members charges were brought of gross violation of the Code of ethics in the matter of professional—or rather unprofessional—advertising.

The evidence was clear and ample.

The member whose case was first brought up, made full confession and apology, asked forgivness and promised unconditional compliance with the code of ethics. After a reprimand, the Society voted to accept his apologies and rely upon his good faith for the future. It was claimed that the offense in this case was committed in part, at least, under a misapprehension of the spirit of the code.

The course pursued by the offender in this instance, is to be commended, and will certainly be to his interest.

In regard to the other two members, the outcome was, to the great regret of all concerned, far different. They did not deny the charges, nor did they attempt a formal defense. They admitted, in the main, what was charged. They made no apologies and no concessions or promises for the future; indeed seemed to manifest a defiant spirit, so that no course was left open for the Society but to expel them, which was promptly done.

Indeed the Society could, under the circumstances, have done nothing less and maintan its self respect, and its connection with the American Dental Association. The occasion was a sad one—one that is much regretted by the best friends of these gentlemen. That one, who for nearly thirty years has enjoyed an untarnished reputation throughout the profession, and the high esteem of all who know him, had made his professional life eminently successful, should voluntarily place himself in a position to be justly expelled from his professional association home is truly lamentable. And but little less is it to be regretted that a young man, having entered the profession with fair prospects and everything bright before him, should assume a position that would draw upon himself the criticism and censure of those whose regard and esteem he ought to prize most highly. But things are often done that cannot be accounted for upon any reasonable hypothesis, and these cases may perhaps be properly placed in that category. All who were present at this meeting seemed to regard it as one of the best ever held by the body. May it hold many more such.

